# Accelerometer-derived physical activity estimation in preschoolers – comparison of cut-point sets incorporating the vector magnitude vs the vertical axis

**DOI:** 10.1186/s12889-019-6837-7

**Published:** 2019-05-06

**Authors:** Claudia S. Leeger-Aschmann, Einat A. Schmutz, Annina E. Zysset, Tanja H. Kakebeeke, Nadine Messerli-Bürgy, Kerstin Stülb, Amar Arhab, Andrea H. Meyer, Simone Munsch, Oskar G. Jenni, Jardena J. Puder, Susi Kriemler

**Affiliations:** 1 Epidemiology, Biostatistics and Prevention Institute, University of Zurich, Hirschengraben 84, 8001 Zurich, Switzerland; 2 Child Development Center, University Children’s Hospital Zurich, Steinwiesstrasse 75, 8032 Zurich, Switzerland; 3 Children’s Research Center, University Children’s Hospital Zurich, Steinwiesstrasse 75, 8032 Zurich, Switzerland; 4 Clinical Child Psychology & Biological Psychology, University of Fribourg, Rue P.A. de Faucigny 2, 1700 Fribourg, Switzerland; 5 Obstetric service, Department Woman-Mother-Child, Lausanne University Hospital, Avenue Pierre Decker 2, 1011 Lausanne, Switzerland; 6 Department of Clinical Psychology and Psychotherapy, University of Fribourg, Rue P.A. de Faucigny 2, 1700 Fribourg, Switzerland; 7 Department of Psychology, Division of Clinical Psychology and Epidemiology, University of Basel, Missionsstrasse 62A, 4055 Basel, Switzerland

**Keywords:** Accelerometry, Behavior, Child, Methodology, Movement, SPLASHY

## Abstract

**Background:**

ActiGraph accelerometers are a widely used tool to objectively measure physical activity (PA) behavior in young children and several validated cut-point sets exist to estimate time spent in different PA intensities (sedentary time, light PA, moderate-to-vigorous PA). Applying different cut-point sets leads to large and meaningful differences in results. So far, only cut-point sets validated for the vertical axis have been compared and only the influence on time spent in moderate-to-vigorous PA has been analyzed.

**Methods:**

A range of validated cut-point sets with their respective epoch length was applied to analyze cross-sectional data of the Swiss Preschoolers’ Health Study (SPLASHY): 1) Vertical axis in combination with an epoch length of 15 s (VA-15), 2) Vertical axis in combination with an epoch length of 60 s (VA-60) and 3) Vector magnitude in combination with an epoch length of 60 s (VM-60). PA was measured for eight consecutive days using ActiGraph accelerometers (wGT3X-BT). Three days were required to be included in the analysis (minimum two weekdays and one weekend-day with at least ten hours recording per day).

**Results:**

Four hundred forty-five preschoolers (mean age 3.9 ± 0.5 years; 46% girls) had valid accelerometer measurements. A longer epoch (VA-60 vs VA-15) resulted in 2% less sedentary time (ST), 18% more light PA (LPA) and 51% less moderate-to-vigorous PA (MVPA); using the vector magnitude compared to the vertical axis (VM-60 vs VA-60) resulted in 34% less ST, 27% more LPA and 63% more MVPA (all *p* ≤ 0.001). Comparing all three sets of cut-points, ST ranged from 4.0 to 6.2 h, LPA from 5.1 to 7.6 h and MVPA from 0.8 to 1.6 h.

**Conclusions:**

Estimated time spent in different PA intensities was strongly influenced by the choice of cut-point sets. Both, axis selection and epoch length need to be considered when comparing different studies especially when they relate PA behavior to health. The differences in the prevalence of children fulfilling PA guidelines highlight the relevance of these findings.

**Trial registration:**

Current Controlled Trials ISRCTN41045021 (date of registration: 21.03.2014).

**Electronic supplementary material:**

The online version of this article (10.1186/s12889-019-6837-7) contains supplementary material, which is available to authorized users.

## Background

Achieving sufficient physical activity (PA) levels in the preschool age is not only important for the healthy development of children [1], but also for establishing movement habits that last throughout adolescence and adulthood [2]. To assess PA behavior in early childhood it is essential to have accurate and objective methods. A reliable and valid tool to objectively measure PA in preschoolers are ActiGraph accelerometers [3]. With these widely used devices the raw acceleration signal is collected at a pre-specified frequency and converted into counts per user-defined time period (epoch length). Age-specific activity thresholds (cut-points) are defined to distinguish sedentary time (ST) vs light PA (LPA) vs moderate-to-vigorous PA (MVPA) [3–7] and may therefore not be valid to assess time spent in certain PA intensities for other age groups due to different PA patterns [8]. These cut-points are validated for a certain combination of axes and epoch lengths. Movement can be measured either on the three-dimensional vector magnitude or only on the vertical axis (also known as axis 1), which is the most frequently used due to the previous lack of availability of the vector magnitude. The vector magnitude incorporates the vertical axis (up-down) as well as the longitudinal (forward-backward) and lateral (left-right) axes. Given the movement patterns characterized by short bouts, it has been argued that short epoch lengths, such as 15 [9], five [8] or even two  seconds [10] should be used to accurately capture PA in this young age group.

Like for older children, there is a large variety of applied cut-point sets in studies analyzing PA behavior in preschoolers [8]. Janssen et al. [11] compared the classification accuracy of six ActiGraph cut-point sets (incorporating the vertical axis and different epoch length) in preschoolers and recommended using the Evenson et al. [6] cut-point to differentiate between ST and LPA and the Pate et al. [3] cut-point for LPA and MVPA. However, using the vertical axis alone may not be appropriate to assess preschoolers’ PA as young children behave in an omnidirectional manner. Despite the fact that three-dimensional ActiGraph devices have been available since 2009, all validation studies except one [4] used only the vertical axis to determine a cut-point set for hip-worn ActiGraph accelerometers [3–7]. There are two other validation studies for three-dimensional ActiGraph cut-points in preschoolers [12, 13] but neither research groups were able to give a complete cut-point set to distinguish between all intensities (ST, LPA, MPA and VPA).

Currently, no gold standard method exists to quantify activity behavior and no agreement has been reached on the most appropriate cut-points for preschool-aged children [14]. The lack of a consensus leads to challenges when comparing and pooling study results, potentially leading to invalid conclusions on the basis of which policy makers define PA guidelines. Applying different cut-point sets is known to create large and significant differences in the estimated time spent in MVPA, ranging from 30 to 260 min/day [15–17], and fulfillment of PA guidelines [18]. However, these studies focused only on the time spent in MVPA and did not investigate the variability in ST or LPA among different cut-point sets. Furthermore, only cut-point sets using the vertical axis were compared and the influence of the vector magnitude was neglected. To address these methodological gaps the aim of this study was to quantify the influence of different cut-point sets on the estimation of time spent in different PA intensities, covering the whole range from ST to MVPA. For this, physical activity estimation in preschoolers was investigated and results of cut-point sets incorporating the vector magnitude vs the vertical axis was compared. As it is known that different factors may influence PA behavior [19, 20] sub-group analyses according to sex, age and weight status can be found in the Additional file 1.

## Methods

### Study design and participants

SPLASHY (Swiss Preschoolers’ Health Study) is a prospective, multi-center cohort study including 555 two to six years old children within Switzerland (ISRCTN41045021). Due to logistical reasons the recruitment and testing of healthy preschoolers took place in childcare centers. 20% of the 639 contacted childcare centers showed first interest, one third of those had to be excluded (mainly due to too few participants), so the final cohort consisted of 84 randomly selected childcare centers from five cantons (Aargau, Bern, Fribourg, Vaud and Zurich) stratified for living area (urban vs rural) and socio-economic region (high vs low) [21]. To obtain a large external validity, exclusion criteria were kept at a minimum; all preschoolers, able to perform the testing (e.g. no motor or cognitive disability), were invited to participate in SPLASHY. The cantonal ethical committee of each study site approved the study protocol and the study was conducted in accordance with the Declaration of Helsinki. Parents gave their written informed consent for study participation and children consented orally.

### Measurements

Anthropometric data were assessed during testing afternoons in the childcare centers. Standing height was measured to the nearest 0.5 cm using a measuring tape. Weight was measured to the nearest 0.1 kg using an electronic scale (Seca, Basel, Switzerland). BMI percentiles were calculated according to World Health Organisation criteria and divided into normal weight (<85th percentile) and overweight (≥85th percentile) [22].

PA was measured for a week with a tri-axial accelerometer (wGT3X-BT, ActiGraph, Pensacola, Florida, USA). The device was attached to the child’s right hip and parents/caregivers received detailed instructions on the use of the activity monitor. They were instructed to wear the monitor during all activities including the night, except for swimming and showering. The accelerometer was programmed to record PA data at a sampling frequency of 30 Hz. Raw data were downloaded using the ActiLife v6.11.4 Firmware v1.0.0, saved as csv-files and further processed by R software (version 3.1.0). For data preparation all non-wear times, defined as time periods of consecutive zero activity counts of 20 min or more in all three axes [9, 23], were excluded. To enable the categorization of the PA intensities according to validated cut-point sets with their specific epoch length, data were aggregated to two versions of expanded epoch lengths of 15 and 60 s. Based on the aggregated counts, the PA intensities were determined using three cut-point sets validated in preschoolers, which differ in axis selection (vertical axis [VA] vs vector magnitude [VM]) and epoch length (15 s vs 60 s): 1) VA-15, 2) VA-60, and 3) VM-60. The cut-points differentiating SB from LPA and LPA from MVPA were 25 and 420 counts per 15 s for VA-15 [11]. The respective cut-points for VA-60 were 240 and 2120 cpm and for VM-60 they were 820 and 3908 cpm. [4]. Due to the lack of validation studies, VM-15 could not be included.

Children aged between three to five years with minimum monitoring of three days (including two weekdays and one weekend-day) with at least 10 hours recording were included in analysis [24, 25]. Only PA data recorded between 7 am and 9 pm were analyzed. SPLASHY had two assessment waves and the first valid PA assessment for each child was taken for analysis. The number of monitoring days, average wear time (h/day), average PA (avPA, cpm), and the average time spent in the different PA intensities (min/day), including sedentary time (ST), light PA (LPA), moderate PA (MPA), moderate-to-vigorous PA (MVPA), vigorous PA (VPA) and any PA (LMVPA = LPA + MVPA) were extracted. The percentage of children fulfilling two widely accepted PA guidelines was used to show the relevance of our findings; the relatively loose PA guideline requesting 180 min LMVPA per day [26–28] and the more stringent one requesting 60 min MVPA per day [18, 29].

### Statistical analysis

Linear multilevel models were applied to compare I) differences of avPA on the three axes (vertical, longitudinal and lateral) and the vector magnitude between both epoch lengths (15 vs 60 s) and II) differences of time spent in various PA intensities (ST, LPA, MPA, MVPA, VPA and LMVPA) when applying different cut-point sets: a) VA-15 vs VA-60, b) VA-60 vs VM-60 and c) VA-15 vs VM-60. Each multilevel model also included wear time (h/day) as a fixed effect and an intercept of the subject’s childcare center as random effect to account for the clustered sampling approach used in this study. Each subject only contributed a single observation to this analysis. Because the models showed light heteroscedasticity (that is, the variance of the outcome increased with larger values of the predictor), we report model results using the so-called “sandwich” estimator of the variance-covariance matrix, which is a more robust estimator of the variance that the usual one. The variance-covariance estimates have been calculated with the R package ‘clubSandwich’ (option type = “CR1S”). The significance level p was set at 0.05 and all models were visually checked for normally distributed residuals using q-q plots. Descriptive statistics for the entire sample are presented in the paper and exploratory subgroup analyses stratified by age (3–3.49 years; 3.5–3.99 years; 4–4.49 years; 4.5–5 years), sex (boys; girls) and weight status (BMI < 85% percentile; BMI ≥ 85% percentile [22]) can be found in the Additional file 1.

## Results

The final sample consisted of 445 preschoolers aged three to five years (mean age 3.9 ± 0.5 years; 54% male). Mean height was 102.5 ± 5.3 cm and mean weight 16.8 ± 2.2 kg; 334 (75.1%) children were categorized as normal weight and 100 (22.5%) as overweight. Average monitoring included 6.0 ± 1.1 days of recordings and mean wear time was 12.8 ± 0.6 h/day. Children with missing PA data did not differ significantly from those included in the analysis according to sex, BMI, living area (urban-rural) or socio-economic state.

### Average counts on different axis

Table 1 shows that avPA varied substantially depending on the axis; avPA assessed by axis 2 (longitudinal, forward-backward) and axis 3 (lateral, left-right) showed higher values than the vertical axis (axis 1, up-down) and therefore had a bigger impact on the three-dimensional vector magnitude. Furthermore, avPA was marginally but significantly higher with the shorter compared to the longer epoch length (15 vs 60 s), in all axes and for the vector magnitude (all *p* ≤ 0.001). Subgroup analyses revealed that the pattern was very similar (see Additional file 1: Tables S2, S3 and S4).

**Table 1:** Mean average physical activity (avPA in cpm) and standard deviation for single axes and the three-dimensional vector magnitude (VM= $\sqrt{{\left(\mathrm{axis}\ 1\right)}^2+{\left(\mathrm{axis}\ 2\right)}^2+{\left(\mathrm{axis}\ 3\right)}^2}$) according to different epoch lengths (15 vs 60 s)

Average PA [cpm]	15 s	60 s
Axis 1	623 ± 152	622 ± 151
Axis 2	800 ± 162	799 ± 161
Axis 3	894 ± 176	892 ± 175
Vector magnitude	1446 ± 281	1409 ± 276

Axis 1 denotes the vertical axis (up-down), axis 2 the longitudinal axis (forward-backward) and axis 3 the lateral axis (left-right)

### Comparison of cut-point sets

**Fig. 1:**
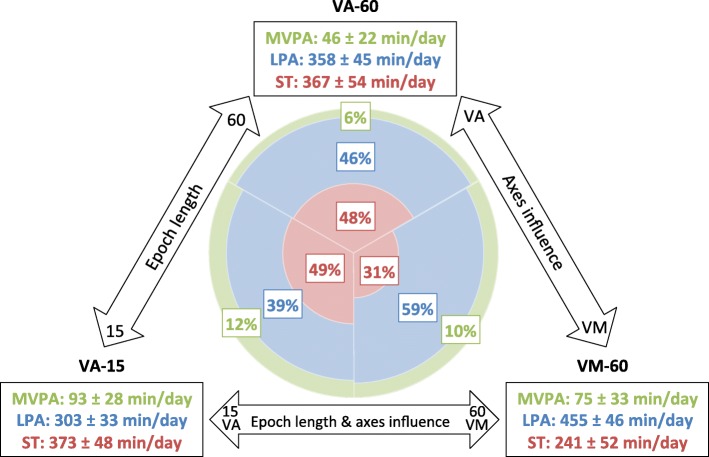
Absolute values (mean ± standard deviation) and percentages of time spent in different PA intensities (ST = sedentary time, LPA = light physical activity and MVPA = moderate-to-vigorous physical activity) according to three different cut-point sets: VA-15 denotes analysis using the vertical axis (VA) with a 15 s epoch length, VA-60 denotes analysis using the vertical axis (VA) with a 60 s epoch length and VM-60 denotes analysis using the vector magnitude (VM) with a 60 s epoch length

Figure 1 shows individual and combined influences of varying epoch length and axis selection based on observed data. A longer epoch length (15 vs 60 s) led to 2% less ST, 18% more LPA and 51% less MVPA. Taking the vector magnitude (vs vertical axis) led to 34% less ST, 27% more LPA and 63% more MVPA. The combined impact of using a longer vs shorter epoch and the vector magnitude instead of vertical axis led to 35% less ST, 51% more LPA and 17% less MVPA. All PA intensities (ST, LPA, MPA, MVPA, VPA, and LMVPA) differed significantly (*p* < 0.001) between the different cut-point sets (VA-15 vs VA-60; VA-60 vs VM-60; VA-15 vs VM-60) except VPA between VA-60 and VM-60, and MPA between VA-15 and VM-60 (both *p* > 0.05; see Additional file 1: Table S5). Subgroup analyses revealed that the pattern and extent of change was strikingly similar (see Additional file 1: Tables S6, S7, S8 and Figure S1 a-h).

The prevalence of children fulfilling PA guidelines was evaluated (average across all valid days): All children fulfilled the recommendation requesting 180 min LMVPA per day; however, when applying the recommendation requesting 60 min MVPA per day, the percentage of preschoolers fulfilling this guideline was strikingly variable ranging from 90% for VA-15, to 22% for VA-60 and 63% for VM-60 (Additional file 1: Table S5). Subgroup analyses revealed very similar results (see Additional file 1: Tables S6, S7 and S8).

## Discussion

The analysis approach of accelerometer-derived data has a huge impact on the outcome. This study showed that avPA was strikingly higher on the longitudinal and lateral axis compared to the commonly used vertical axis, but the influence of shorter compared to longer epoch length was marginal. Furthermore, time spent at different PA intensities varied substantially depending on the accelerometer cut-point set applied (VA-15, VA-60 and VM-60). This variation challenges the accurate depiction of PA and should be taken into consideration when developing PA guidelines.

### Average counts on different axes

For avPA, 28 and 43% higher values were detected on the longitudinal and lateral axes respectively, compared to the commonly used vertical axis (Tab. 1) suggesting that simply taking the vertical axis as representative may not be valid to represent the PA behavior of each child. In children aged five to nine years, Jimmy et al. [30] also found varying avPA values for the different axes, which were dependent on specific activities: Walking activities and jogging resulted in the highest counts on the vertical axis but activities like playing with toy trains, free play and soccer led to more counts on the longitudinal and lateral axes than on the vertical axis. Our finding of varying activity counts among individual axes thus supports the use of the vector magnitude, which takes all axes of the three-dimensional system into account. Although avPA was significantly higher with shorter compared to longer epoch length, differences within axis were marginal and were therefore not clinically relevant. This was most likely due to the high co-linearity of the two variables and the integration over a longer interval, leading to smoothing of extreme values.

### Comparison of cut-point sets

The results regarding time spent in different PA intensities differed tremendously depending on the applied cut-point set (Fig. 1): **a) Effect of epoch lengths (VA-15 vs VA-60):** A longer epoch length captured less MVPA because the behavior was classified as LPA; ST was only marginally influenced. Even though experts [8–10] postulate that shorter epoch lengths are better, preferably 15 s or less, due to preschoolers activity patterns in short bursts [10, 14], there is generally weak evidence to support this idea according to systematic reviews that correlated PA intensities and health in preschoolers [1]. Previous research showed that shorter epoch length capture more PA, despite converting the cut-points by dividing or multiplying to fit different epoch lengths; i.e. if the cut-point for 15 s epoch is 25 counts, it is 100 counts for an epoch length of 60 s [17, 31, 32]. However, several authors recommend using the same epoch length like during the validation study [8, 9, 14]. Additionally one should be aware that even reintegrating ActiGraph measurement with a short epoch length in longer epoch length results in more PA output compared to the recording with the respective longer epoch length [17]. **b) Effect of axis selection (VA-60 vs VM-60):** When using the vector magnitude (vs the vertical axis), children were categorized as being more active; they showed less ST, and more LPA and MVPA. This finding may not be surprising as the VM-60 cut-point set not only takes the movement on the vertical axis but also those on the longitudinal and lateral axes into account. A study in seniors showed similar results, as more LPA and MVPA was recorded when cut-points for the vector magnitude instead the vertical axis were applied [33]. Another study with children aged five to nine years concluded that their cut-points based on the vector magnitude did not appear to reflect the intensity categories more accurately than cut-points based on the vertical axis [34]. However, a very short epoch length of five seconds was used for calibration, which is known to be more sensitive to capturing high intensity activity than longer epoch lengths [17]. **c) Combined effect of epoch length and vector selection (VA-15 vs VM-60):** The combined impact of a longer epoch and vector magnitude led to less ST, more LPA and less MVPA. Although we cannot resolve which cut-point set is more appropriate, from a behavioral perspective considering preschoolers’ omnidirectional activity pattern in short bursts, the use of a three-dimensional system with a short epoch length makes logical sense to assess their PA behavior. Unfortunately, validation studies for this age group are still missing.

### General thoughts

Our study is not the first comparing different validated ActiGraph cut-point sets and detecting the discrepancy in time spent in activity levels in preschoolers [14–17] and school-aged children [31, 35, 36]. Novel is that we looked at the effect of preschoolers’ omni-directional movement behavior (e.g. vector magnitude vs single vertical axis) and focused on the whole range of PA behavior (ST, LPA and MVPA) rather than only at MVPA. The use of a wide variety of cut-point sets generates disparity in PA estimates leading to lack in comparability [15–17]. As a solution to this cut-point non-equivalence, some authors developed formulas, which convert PA estimates from one set of cut-points into estimates from another set of cut-point [37, 38]. Although these conversion formulas may facilitate comparisons across studies, they do not answer the question of which cut-points are most appropriate for the preschool population. Like previous authors [14, 35] we request a consensus about a common approach to analyze PA behavior by accelerometers. This will only be possible through additional series of calibration and independent validation studies. If we stay in this conventional system using company-based software for analyses, priorities should be given to validation of cut-point sets combining a short epoch length of 15 s or less with the three-dimensional VM, as this combination best reflect the natural PA behavior of preschoolers. Alternatively, we may agree on moving back to the use and documentation of raw acceleration signals, rather than proprietary counts, as proposed by a recent critical and elegant paper [39].

The ability to accurately estimate PA of young children is necessary to make well-informed decisions and potential recommendations for public health policies. The proportion of children engaging in the recommended 60 min of MVPA per day, that ranged from 22 to 90% depending on the set of cut-points applied, reflects the relevance of this statement. Despite ample evidence that adult diseases have their origins in childhood [40], evidence on the link between preschool PA and health outcomes is scarce [1]. Obviously preschoolers are generally healthy and non-communicable diseases develop much later and over decades, making the link between PA and health outcomes at this young age a true challenge. A better understanding of the amount, frequency and intensity of young children’s PA for persistent health benefits is needed, as established for older children [41, 42]. This can only be reached by focusing on long-term cohorts that are able to relate PA behavior at preschool age with relevant health outcomes later in life. In the meantime comparability of study results can be reached by reporting the cut-point set independent avPA (in cpm) and using conversion formulas as a tool to compare the PA behavior among studies.

### Strengths and limitations

A strength of our study is the relatively large and randomly selected sample of preschoolers with a reliable and objective PA assessment. Furthermore, cut-point sets covering the whole range of PA intensities and validated for the newest ActiGraph generation (GT3X) in very similar age groups were studied. The chosen cut-point sets varied not only according to the epoch length but also axis used, showing the additional effect of measurement dimensions. Limitations of our study include that the study participation was voluntary and focused on children attending childcare centers, which may have led to a potential participation bias. Our analysis approach included a number of data selection decisions (number of days, length of day, and definition of non-wear time) and any of these decisions could have influenced the results. There are several constraints concerning measurement of PA by accelerometers such as imprecise assessments of rolling activities like riding bogie wheels and the incapability to measure water activities, which both could have led to an underestimation of PA. Even though nighttime sleep between 9 pm and 7 am was removed, most of the preschoolers still took afternoon naps; this daytime sleep could have been measured incorrectly. Yet, all these limitations were true for all different versions of analyses, therefore any bias is expected to be equal among all applied cut-point sets.

## Conclusions

The analysis of objectively assessed PA behavior of preschoolers is influenced by various factors. I) Average PA counts were strikingly higher on the longitudinal and lateral axis compared to the generally used vertical axis. This supports the use of the vector magnitude that takes all axes of the three-dimensional system into account. II) The choice of accelerometer cut-point set had a substantial impact on measured time spent in different PA intensities. Both, the epoch length and the choice of axis have to be considered when comparing different studies and may explain part of the differences in observed PA behavior. More validation studies that best reflect PA behavior of preschoolers (three-dimensional VM and short epoch length) are required. Additionally, more long-term research, able to relate PA behavior of preschoolers to health outcomes in later life, is needed. Meanwhile it is important to report not only the time spent in certain activity levels but also the cut-point independent avPA (in cpm) or raw acceleration signals to analyze amount and intensity of PA behavior to improve comparability between study results.

## Additional file

Additional file 1: Additional tables and subgroup analyses according to age, sex and weight status are summarized in the *additional file of “Accelerometer-derived physical activity estimation in preschoolers – Comparison of cut-point sets incorporating the vector magnitude vs the vertical axis”*. (PDF 592 kb)

## Abbreviations

avPA: Average physical activity [cpm]
BMI: Body mass index
cpm: Count per minute
h/day: Hours per day
LMVPA: Any physical activity (LMVPA = LPA + MVPA) [min/day]
LPA: Light physical activity [min/day]
min/day: Minutes per day
MPA: Moderate physical activity [min/day]
MVPA: Moderate-to-vigorous physical activity [min/day]
PA: Physical activity
SPLASHY: Swiss Preschoolers’ Health Study
ST: Sedentary time [min/day]
VA: Vertical axis
VM: Vector magnitude
VPA: Vigorous physical activity [min/day]
