# Supplementary material for: Accelerometer-derived physical activity estimation in preschoolers – comparison of cut-point sets incorporating the vector magnitude vs the vertical axis
Source: BMC Public Health. 2019 May 6;19:513. doi: 10.1186/s12889-019-6837-7 (PMC6501292; doi:10.1186/s12889-019-6837-7)

Additional file of "Accelerometer-derived physical activity estimation in preschoolers – Comparison of cut-point sets incorporating the vector magnitude vs the vertical axis"

Table S1 a-c. Characteristics of the study population according to (a) age groups (3-3.49 years; 3.5-3.99 years; 4-4.49 years; 4.5-5 years), b) gender (boys; girls) and c) weight status (BMI<85th percentile; BMI ≥ 85th percentile). Results are displayed either as means ± standard deviation or as absolute values with relative percentages in brackets.

a)

**Age group**	**3-3.49 years**	**3.5-3.99 years**	**4-4.49 years**	**4.5-5 years**
**Sample size**	122	143	106	74
**Age (y)**	3.3±0.1	3.7±0.1	4.2±0.2	4.7±0.1
**Sex male-female (%)**	62-60 (51-49)	80-63 (56-44)	58-48 (55-45)	39-35 (53-47)
**Height (cm)**	98.1±3.5	101.6±4.0	104.7±4.3	108.2±4.5
**Weight (kg)**	15.7±1.9	16.5±1.8	17.4±2.0	18.4±2.3
**BMI normal-overweight (%)**	85-35 (70-29)	111-28 (78-20)	83-19 (78-18)	55-18 (74-24)
**Wear-time (h/day)**	12.8±0.6	12.9±0.5	12.9±0.6	13.0±0.7
**Number of monitoring days**	6.0±1.1	6.0±0.9	6.0±0.9	5.9±0.8

b)

**Gender**	**Boys**	**Girls**
**Sample size**	239	206
**Age (y)**	3.9±0.5	3.9±0.5
**Height (cm)**	102.9±5.7	102.0±4.9
**Weight (kg)**	17.0±2.2	16.6±2.1
**BMI normal-overweight (%)**	182-51 (76-21)	152-49 (74-24)
**Wear-time (h/day)**	12.9±0.6	12.8±0.6
**Number of monitoring days**	6.0±1.3	6.0±0.8

c)

**Weight status**	**Normal weight**	**Overweight**
**Sample size**	334	100
**Age (y)**	3.9±0.5	3.8±0.6
**Sex male-female (%)**	182-152 (54-46)	51-49 (51-49)
**Height (cm)**	102.5±5.1	102.5±4.5
**Weight (kg)**	16.2±1.7	18.8±2.4
**Wear-time (h/day)**	12.9±0.6	12.8±0.6
**Number of monitoring days**	6.0±1.1	5.9±1.0

Table S2 a & b. Average physical activity (avPA; mean ± standard deviation) for different age groups for each axis and the vector magnitude (VM = $\sqrt{(\text{axis }1)^2 + (\text{axis }2)^2 + (\text{axis }3)^2}$). Axis 1 is the vertical axis (VA, up and down), axis 2 is the longitudinal axis (for- and backward) and axis 3 is the lateral axis (left and right).

a)

15sec [cpm]	3-3.49 years	3.5-3.99 years	4-4.49 years	4.5-5 years
**Axis 1**	593±127	622±165	644±145	647±164
**Axis 2**	774±145	804±171	816±141	814±191
**Axis 3**	844±161	893±185	924±164	937±180
**Vector magnitude**	1380±250	1447+302	1488±253	1496±308

b)

60sec [cpm]	3-3.49 years	3.5-3.99 years	4-4.49 years	4.5-5 years
**Axis 1**	591±125	620±165	642±146	646±164
**Axis 2**	772±144	802±170	814±141	812±191
**Axis 3**	842±160	891±184	921±164	935±180
**Vector magnitude**	1345±242	1409±297	1448±249	1459±304

Table S3 a & b. Average physical activity (avPA; means ± standard deviation) for boys and girls for each axis and the vector magnitude (VM = $\sqrt{(\text{axis } 1)^2 + (\text{axis } 2)^2 + (\text{axis } 3)^2}$). Axis 1 is the vertical axis (VA, up and down), axis 2 is the longitudinal axis (for- and backward) and axis 3 is the lateral axis (left and right).

a)

15sec [cpm]	Boys	Girls
**Axis 1**	648±164	595±131
**Axis 2**	827±162	769±156
**Axis 3**	917±185	868±162
**Vector magnitude**	1489±295	1397±256

b)

60sec [cpm]	Boys	Girls
**Axis 1**	646±163	593±130
**Axis 2**	824±161	768±155
**Axis 3**	915±184	866±161
**Vector magnitude**	1451±290	1361±250

Table S4 a & b. Average physical activity (avPA; means ± standard deviation) for children categorized as normal weight (BMI<85th percentile) and overweight (BMI ≥ 85th percentile) for each axis and the vector magnitude (VM = $\sqrt{(\text{axis } 1)^2 + (\text{axis } 2)^2 + (\text{axis } 3)^2}$). Axis 1 is the vertical axis (VA, up and down), axis 2 is the longitudinal axis (for- and backward) and axis 3 is the lateral axis (left and right).

a)

**15sec [cpm]**	**Normal weight**	**Overweight**
**Axis 1**	617±135	638±162
**Axis 2**	797±151	806±179
**Axis 3**	894±164	885±186
**Vector magnitude**	1440±257	1453±302

b)

**60sec [cpm]**	**Normal weight**	**Overweight**
**Axis 1**	615±135	636±161
**Axis 2**	795±150	804±178
**Axis 3**	892±164	882±185
**Vector magnitude**	1403±252	1414±295

Table S5. Mean time spent in different physical activity intensities (ST = Sedentary Time, LPA = Light Physical Activity, MPA = Moderate Physical Activity, VPA = Vigorous Physical activity, MVPA = Moderate-to-Vigorous Physical Activity and any physical activity (LMVPA = LPA+MVPA)) according to three different PA analysis approaches: Vertical Axis-15sec epoch (VA-15), Vertical Axis-60sec epoch (VA-60) and Vector Magnitude-60sec epoch (VM-60). Moreover, the table shows how many children fulfil two physical activity guidelines requesting either three hours any physical activity (LMVPA) or one hour MVPA. Results are displayed either as mean ± standard deviation or as absolute values with relative percentages in brackets.
All PA intensities (ST, LPA, MPA, MVPA, VPA, and LMVPA) differed highly significantly ($p<0.001$) between the different cut-point sets (VA-15 vs VA-60; VA-60 vs VM-60; VA-15 vs VM-60) except VPA between VA-60 and VM-60 and MPA between VA-15 and VM-60 (both $p>0.05$). The cut-points for VPA were 842 counts per 15 seconds for VA-15, 4450 cpm for VA-60 and 6112 cpm for VM-60.

**All Kids (n=445)**	**VA-15**	**VA-60**	**VM-60**
**ST (min/day)**	373 ± 48	367 ± 54	240 ± 52
**LPA (min/day)**	303 ± 33	358 ± 45	455 ± 46
**MPA (min/day)**	71 ± 19	41 ± 19	70 ± 30
**VPA (min/day)**	22 ± 11	5 ± 5	5 ± 6
**MVPA (min/day)**	93 ± 28	45 ± 22	75 ± 33
**LMVPA (min/day)**	396 ± 50	404 ± 55	530 ± 58
**Children fulfilling PA guidelines 180min<LMVPA (%)**	445 (100)	445 (100)	445 (100)
**Children fulfilling PA guidelines 60min<MVPA (%)**	399 (90)	100 (22)	280 (63)

Table S6 a-c. Mean time spent in different PA intensities (ST = Sedentary Time, LPA = Light Physical Activity, MPA = Moderate Physical Activity, VPA = Vigorous Physical activity, MVPA = Moderate-to-Vigorous Physical Activity and any physical activity (LMVPA = LPA+MVPA)) according to three different PA analysis approaches (a) VA-15 = Vertical Axis-15sec epoch, b) VA-60 = Vertical Axis-60sec epoch and c) VM-60 = Vector Magnitude-60sec epoch) separately for different age groups. Moreover, the table shows how many children fulfil two physical activity guidelines requesting either three hours any physical activity (LMVPA) or one hour MVPA. Results are displayed either as mean ± standard deviation or as absolute values with relative percentages in brackets. The cut-points for VPA were 842 counts per 15 seconds for VA-15, 4450 cpm for VA-60 and 6112 cpm for VM-60.

a)

**VA-15**	**3-3.49 years**	**3.5-3.99 years**	**4-4.49 years**	**4.5-5 years**
**ST (min/day)**	370±45	375±50	371±49	377±49
**LPA (min/day)**	302±32	301±35	306±32	304±34
**MPA (min/day)**	66±17	71±19	74±19	73±19
**VPA (min/day)**	19±9	22±11	23±10	25±14
**MVPA (min/day)**	85±26	93±28	98±28	98±30
**LMVPA (min/day)**	388±47	394±52	401±49	402±51
**Children fulfilling PA guidelines 180min<LMVPA (%)**	122 (100)	143 (100)	106 (100)	74 (100)
**Children fulfilling PA guidelines 60min<MVPA (%)**	104 (85)	130 (91)	99 (93)	66 (89)

b)

**VA-60**	**3-3.49 years**	**3.5-3.99 years**	**4-4.49 years**	**4.5-5 years**
**ST (min/day)**	366±50	368±55	364±55	375±55
**LPA (min/day)**	354±44	357±46	364±43	360±45
**MPA (min/day)**	35±17	41±19	44±20	45±22
**VPA (min/day)**	4±4	5±6	5±5	6±7
**MVPA (min/day)**	40±19	45±22	49±22	51±26
**LMVPA (min/day)**	394±53	402±57	413±54	410±56
**Children fulfilling PA guidelines 180min<LMVPA (%)**	122 (100)	143 (100)	106 (100)	74 (100)
**Children fulfilling PA guidelines 60min<MVPA (%)**	21 (17)	29 (20)	30 (28)	20 (27)

c)

**VM-60**	**3-3.49 years**	**3.5-3.99 years**	**4-4.49 years**	**4.5-5 years**
**ST (min/day)**	247±51	243±52	235±54	235±50
**LPA (min/day)**	449±47	453±44	461±47	461±46
**MPA (min/day)**	61±28	70±30*	75±29	78±30
**VPA (min/day)**	4±4	5±6	5±5	7±7
**MVPA (min/day)**	65±30	74±33*	81±31	85±35
**LMVPA (min/day)**	513±57	528±55	542±58	546±56
**Children fulfilling PA guidelines 180min<LMVPA (%)**	122 (100)	143 (100)	106 (100)	74 (100)
**Children fulfilling PA guidelines 60min<MVPA (%)**	59 (48)	88 (62)	78 (73)	55 (74)

Table S7 a-c. Mean time spent in different PA intensities (ST = Sedentary Time, LPA = Light Physical Activity, MPA = Moderate Physical Activity, VPA = Vigorous Physical activity, MVPA = Moderate-to-Vigorous Physical Activity and any physical activity (LMVPA = LPA+MVPA)) according to three different PA analysis approaches (a) VA-15 = Vertical Axis-15sec epoch, b) VA-60 = Vertical Axis-60sec epoch and c) VM-60 = Vector Magnitude-60sec epoch) separately for boys and girls. Moreover the table shows how many children fulfil two physical activity guidelines requesting either three hours any physical activity (LMVPA) or one hour MVPA. Results are displayed either as mean ± standard deviation or as absolute values with relative percentages in brackets. The cut-points for VPA were 842 counts per 15 seconds for VA-15, 4450 cpm for VA-60 and 6112 cpm for VM-60.

a)

VA-15	Boys	Girls
ST (min/day)	370±47	377±49
LPA (min/day)	303±33	303±33
MPA (min/day)	76±19	65±17
VPA (min/day)	24±13	20±8
MVPA (min/day)	100±30	85±24
LMVPA (min/day)	403±50	388±49
Children fulfilling PA guidelines 180min<LMVPA (%)	239 (100)	206 (100)
Children fulfilling PA guidelines 60min<MVPA (%)	222 (93)	177 (86)

b)

VA-60	Boys	Girls
ST (min/day)	364±53	371±54
LPA (min/day)	360±43	357±46
MPA (min/day)	46±21	34±15
VPA (min/day)	5±6	5±5
MVPA (min/day)	51±24	39±17
LMVPA (min/day)	410±55	396±54
Children fulfilling PA guidelines 180min<LMVPA (%)	239 (100)	206 (100)
Children fulfilling PA guidelines 60min<MVPA (%)	74 (31)	26 (13)

c)

VM-60	Boys	Girls
ST (min/day)	242±51	239±53
LPA (min/day)	450±45	460±47
MPA (min/day)	77±30	62±27
VPA (min/day)	5±6	5±4
MVPA (min/day)	82±34	67±29
LMVPA (min/day)	532±57	528±59
Children fulfilling PA guidelines 180min<LMVPA (%)	239 (100)	206 (100)
Children fulfilling PA guidelines 60min<MVPA (%)	167 (70)	113 (55)

Table S8 a-c. Mean Time spent in different PA intensities (ST = Sedentary Time, LPA = Light Physical Activity, MPA = Moderate Physical Activity, VPA = Vigorous Physical activity, MVPA = Moderate-to-Vigorous Physical Activity and any physical activity (LMVPA = LPA+MVPA)) according to three different PA analysis approaches (a) VA-15 = Vertical Axis-15sec epoch, b) VA-60 = Vertical Axis-60sec epoch and c) VM-60 = Vector Magnitude-60sec epoch) separately for children categorized as normal weight (BMI<85th percentile) and overweight (BMI≥85th percentile). Moreover, the table shows how many children fulfil two physical activity guidelines requesting either three hours any physical activity (LMVPA) or one hour MVPA. Results are displayed either as mean ± standard deviation or as absolute values with relative percentages in brackets. The cut-points for VPA were 842 counts per 15 seconds for VA-15, 4450 cpm for VA-60 and 6112 cpm for VM-60.

a)

**VA-15**	**Normal weight**	**Overweight**
**ST (min/day)**	375±47	366±49
**LPA (min/day)**	303±33	302±34
**MPA (min/day)**	70±19	72±19
**VPA (min/day)**	21±10	24±13
**MVPA (min/day)**	91±27	96±31
**LMVPA (min/day)**	395±50	398±51
**Children fulfilling PA guidelines 180min<LMVPA (%)**	334 (100)	100 (100)
**Children fulfilling PA guidelines 60min<MVPA (%)**	299 (90)	90 (90)

b)

**VA-60**	**Normal weight**	**Overweight**
**ST (min/day)**	369±53	358±54
**LPA (min/day)**	358±45	360±44
**MPA (min/day)**	40±18	42±23
**VPA (min/day)**	5±4	5±6
**MVPA (min/day)**	44±20	48±26
**LMVPA (min/day)**	403±55	407±56
**Children fulfilling PA guidelines 180min<LMVPA (%)**	334 (100)	100 (100)
**Children fulfilling PA guidelines 60min<MVPA (%)**	73 (22)	25 (25)

c)

**VM-60**	**Normal weight**	**Overweight**
**ST (min/day)**	241±51	241±53
**LPA (min/day)**	456±44	451±51
**MPA (min/day)**	71±29	68±32
**VPA (min/day)**	5±4	5±7
**MVPA (min/day)**	75±31	73±36
**LMVPA (min/day)**	532±57	524±59
**Children fulfilling PA guidelines 180min<LMVPA (%)**	334 (100)	100 (100)
**Children fulfilling PA guidelines 60min<MVPA (%)**	216 (65)	56 (56)

Figure S1 a-h. Time spent in different PA intensities (ST = sedentary time, LPA = light physical activity and MVPA = moderate-to-vigorous physical activity) of an average day (100% is between 12.8 h/day for children younger than 3.5 years to 13.0 h/day for children aged over 4.5 years) according to three different PA analysis approaches (VA-15 = Vertical Axis-15sec epoch, VA-60 = Vertical Axis-60sec epoch and VM-60 = Vector Magnitude-60sec epoch).
Subgroup analyses according to age groups (a-d), sex (e-f) and weight status (g-h) revealed that the pattern and extent of change was strikingly similar for any subgroup: with VA-15 children showed 48-49% ST, 39-40% LPA and 11-13% MVPA; with VA-60 47-48% ST, 46-47% LPA and 5-7% MVPA; and with VM-60 30-32% ST, 58-60% LPA and 9-11% MVPA (all results are wear-time adjusted).

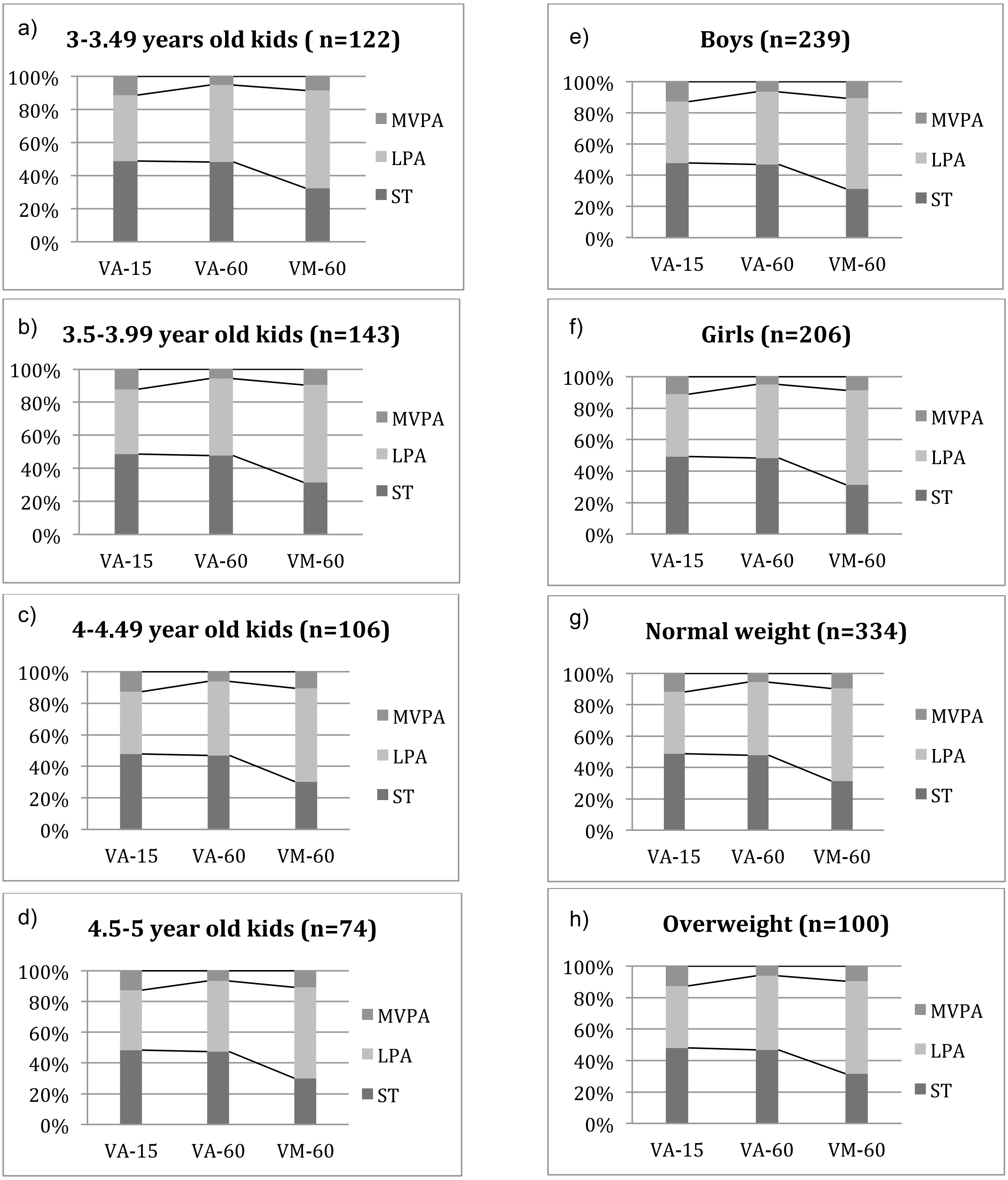

Supplement: Supplementary file 1 — Additional tables and subgroup analyses according to age, sex and weight status are summarized in the additional file of “Accelerometer-derived physical activity estimation in preschoolers – Comparison of cut-point sets incorporating the vector magnitude vs the vertical axis”. (PDF 592 kb) [file 12889_2019_6837_MOESM1_ESM.pdf]
